# Ischemic stroke following a wasp sting – a rare complication: a case report

**DOI:** 10.1186/s13256-018-1839-0

**Published:** 2018-10-14

**Authors:** Chamara Dalugama, Indika Bandara Gawarammana

**Affiliations:** Department of Medicine, University of Peradeniya, Peradeniya, Sri Lanka

**Keywords:** Wasp sting, Ischemic stroke, Vascular

## Abstract

**Background:**

Wasp stings are commonly encountered worldwide and result in a variety of clinical manifestations including local and systemic reactions. Neurological and vascular complications are rarely reported following a wasp sting.

**Case presentation:**

A 69-year-old Sri Lankan Tamil man presented to our hospital with focal neurological deficit following multiple wasp stings; the deficit was confirmed to be an acute infarction on magnetic resonance imaging scan. He was screened for metabolic risk factors which were negative and he had a normal two-dimensional echocardiogram and normal carotid arteries in carotid duplex which excluded potential sources of thromboembolism.

**Conclusion:**

Treating physicians should be aware of the rare but possible complication of ischemic stroke following a case of wasp sting.

## Background

Wasp stings are commonly encountered worldwide. They can result in a variety of clinical manifestations of which local reactions are the commonest [1]. Neurological and vascular manifestations, including ischemic strokes [2–4], are very rarely reported following wasp sting. The pathophysiology of ischemic stroke following wasp sting is probably due to vasoactive, inflammatory and thrombogenic properties of wasp venom [5–7]. An alternative mechanism may be vascular spasms caused by venom [8–10]. We report a previously healthy Sri Lankan man presenting with an ischemic stroke following a wasp sting.

## Case presentation

We report the case of a 69-year-old Sri Lankan Tamil man from Kandy who presented to the toxicology unit following a wasp sting. He was stung by three wasps in the evening and was brought to the toxicology unit of Teaching Hospital, Peradeniya with acute onset slurring of speech, deviation of mouth to the left side, with right-side weakness of his body. He had mild local reaction at the sites of wasp sting, but no anaphylaxis. He was apparently well before this event without any chronic medical illnesses.

On admission to the toxicology unit he was conscious and rational. He was neither pale nor plethoric. He had a regular pulse of 80 beats per minute and blood pressure of 140/90 mmHg. On clinical examination he had no cardiac murmurs or carotid bruits. He had expressive aphasia, right-side upper motor neuron-type facial nerve palsy, with grade four weakness of the right side of his body.

**Fig. 1:**
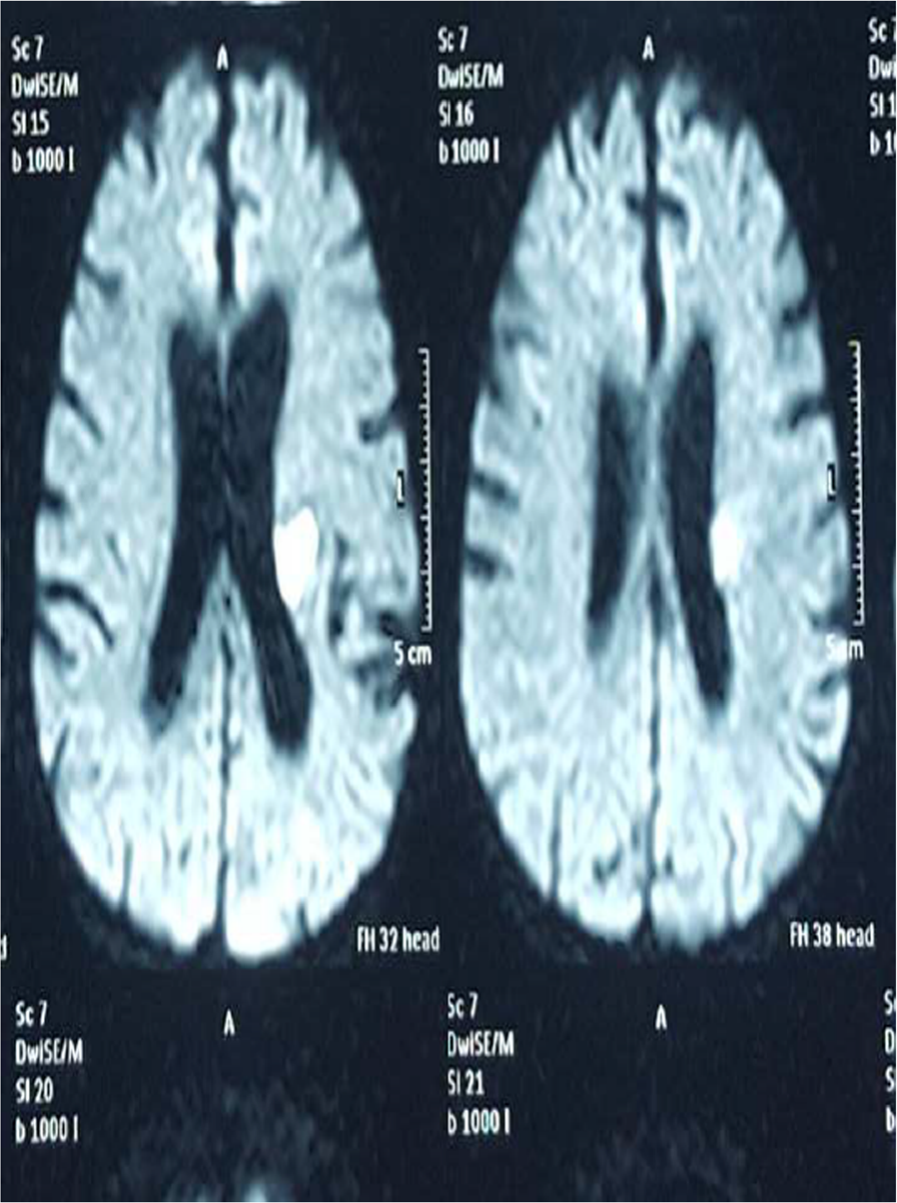
Magnetic resonance imaging showing an acute infarction in the left posterior frontal white matter

Random blood sugar on admission was 121 mg/dL. Magnetic resonance imaging (MRI) of his brain revealed an acute infarction in the left posterior frontal white matter, which was compatible with the clinical presentation (Fig. 1). His complete blood count showed hemoglobin of 13.6 g/dL and platelet count of 350 × 10^9^/L. Clotting profile was within normal limits. An electrocardiogram (ECG) was in sinus rhythm and two-dimensional echocardiogram was normal with good left ventricular systolic function. Carotid duplex showed anatomically normal carotid arteries. Fasting blood sugar was 4 mmol/L. Total cholesterol was 148 mg/dL (< 180) and low-density lipoprotein (LDL) cholesterol was 90 mg/dL (< 130). His erythrocyte sedimentation rate was 11 mm in first hour. His renal functions were normal.

**Fig. 2:**
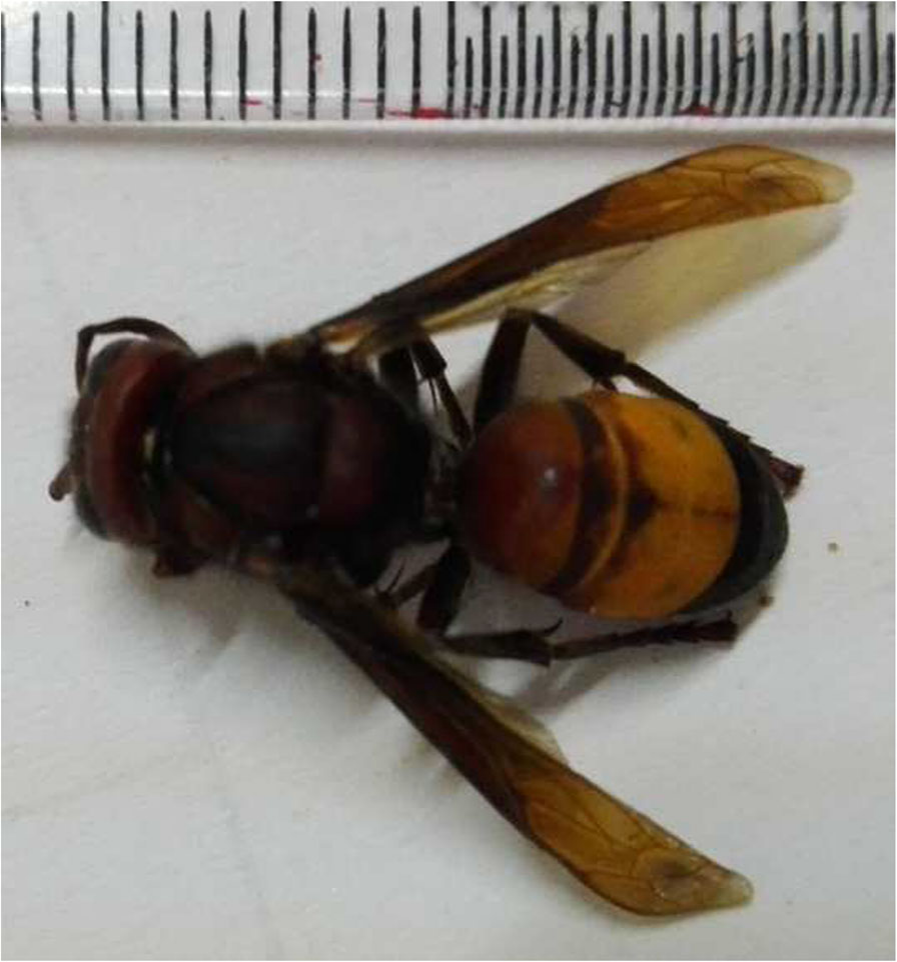
The wasp – *Vespa tropica*

He was started on aspirin and atorvastatin. Rehabilitation was arranged with physiotherapy and speech therapy. His place of residence was visited by the authors and the members of the wasp species were found and identified as *Vespa tropica* of family Vespidae (Fig. 2). On discharge, he had a mild right facial droop but normal strength in his right arm and leg.

## Discussion

Wasps are members of the order Hymenoptera, suborder Apocrita [1]. Wasp sting events have occurred worldwide, especially in developing countries with swarm attack. Despite the common occurrence of insect stings and local and systemic allergic reactions [1], there are few reports of vascular complications such as myocardial infarction or stroke following wasp stings.

Our case reports a previously healthy man developing an acute stroke following a wasp sting. This case report clearly shows that as few as three wasp stings can give rise to ischemic cerebral infarctions. It is unlikely that the cause of ischemic stroke is due to anaphylaxis-induced hypotension as suggested by the territory. As our patient had no known predisposing causes of strokes, it is extremely likely that the wasp sting caused the event in a previously healthy vascular anatomy.

Few cases of cerebral infarction following wasp sting have been reported in the literature. However, the species has not been positively identified in any of the cases. In the case reported by Crawley *et al.* [2], the victim had been hypotensive for a period of time and had a prolonged stay in an intensive care unit. The cause of the occipital lobe infarction may have been due to many other known risk factors. In another case report, three wasp stings were followed by collapse and tonic-clonic seizure [3]. There was no recoded hypotension. The patient was treated with adrenaline, steroids, and phenobarbitone and later found to have hemiparesis and computed tomography (CT) confirmed cerebral infarction. In another two case reports the patients died after wasp sting and at postmortem cerebral infarctions were found [4]. In the latter two cases there was no positive identification of the species and there was no mention of other known risk factors for ischemic strokes. We have excluded common risk factors of ischemic strokes and it is likely that the toxins present in the venom are the most likely cause of ischemic stroke.

Wasp venom contains a wide array of amines, peptides, and enzymes that are responsible for local and systemic reactions. The first set of information on wasp venom at a molecular level was given in the study by Sookrung *et al.* [5], which studied the venom of *Vespa affinis* and classified the venom into typical venom components, and structural and housekeeping proteins. The venom of *Vespa affinis* and *Vespa tropica* appear to be similar. The pathophysiology of ischemic infarctions following wasp sting is yet to be described. Wasp venom contains vasoactive, inflammatory, and thrombogenic peptides and amines including histamines, leukotrienes, and thromboxane. Leukotrienes and thromboxane cause platelet aggregation and vasoconstriction [6]. Direct toxic effects are mediated by mixtures of low molecular weight compounds such as serotonin, histamine, acetylcholine, and several kinins [6]. Phospholipases in wasp venom can elicit an immunoglobulin E response resulting in mast cell activation which will in turn release histamine and lead to the release of a cascade of *de novo* synthesized chemical mediators [7].

An alternative mechanism may be vasospasm. In the literature there are few cases of myocardial infarction occurring after bee sting has been reported. Massing *et al.* [8] reported a case of inferior myocardial infarction in a young man following bee sting. He was followed up with a coronary angiogram after the acute phase and his coronary arteries were angiographically healthy. Valla *et al.* [9] also reported myocardial infarction in a 45-year-old man following an anaphylactic reaction to a wasp sting. Ceyhan *et al.* [10] reported a non-ST elevation acute myocardial infarction in a patient envenomed by a bee sting. However, in these instances a coronary angiogram revealed healthy non-occluded coronary arteries and probably the initial myocardial injury was caused by vasospasm. Similar phenomena can operate in the cerebral circulation and can cause acute focal neurological deficits. However, irreversible neurological deficit makes it unlikely in our case.

The ischemic stroke in our patient could be attributed to wasp venom which is vasoactive, inflammatory, and thrombogenic.

## Conclusions

Physicians encounter large numbers of hymenopteran sting cases each year. These patients typically present with local reactions. Systemic manifestations, such as anaphylaxis, are less common. Neurological manifestations are extremely rare. Treating physicians should be aware of the rare but possible complication of ischemic stroke in a case of wasp sting.

## Abbreviations

CT: Computed tomography
ECG: Electrocardiogram
LDL: Low-density lipoprotein
MRI: Magnetic resonance imaging
